# The Driving Waveform Design Method of Power-Law Fluid Piezoelectric Printing Based on Iterative Learning Control

**DOI:** 10.3390/s22030935

**Published:** 2022-01-25

**Authors:** Ju Peng, Jin Huang, Jianjun Wang, Fanbo Meng, Hongxiao Gong, Bu Ping

**Affiliations:** Key Laboratory of Electronic Equipment Structure Design of Ministry of Education, Xidian University, Xi’an 710071, China; pengju@stu.xidian.edu.cn (J.P.); wangjianjun@xidian.edu.cn (J.W.); fbmeng@xidian.edu.cn (F.M.); hxgong@stu.xidian.edu.cn (H.G.); pingbu@stu.xidian.edu.cn (B.P.)

**Keywords:** piezoelectric three-dimensional inkjet printing, driving waveform design, iterative learning control

## Abstract

In some applications of piezoelectric three-dimensional inkjet printing, the materials used are power-law fluids as they are shear thinning. Their time-varying viscosities affect the droplet formation, which is determined by the volume flow rate at the nozzle outlet. To obtain a fine printing effect, it is necessary to present a driving waveform design method that considers the shear-thinning viscosities of materials to control the volume flow rate at the nozzle outlet, which lays the foundation for the single and stable droplet generation during the printing process. In this research, we established the relationship between the driving waveform and the volume flow rate at the nozzle outlet by modifying a model that describes the inkjet mechanism of power-law fluid. The modified model was used to present a driving waveform design method based on iterative learning control. The iterative learning law of the method was designed based on the gradient descent algorithm and demonstrated its convergence. The driving waveform design method was verified to be practical and feasible by implementing drop generation experiments.

## 1. Introduction

Inkjet-based 3D printing is the process that generates single and stable droplets and deposits the droplets on the substrate to form a three-dimensional structure. With the characteristics of high compatibility, this technology has broad application perspective in the fields of machinery [1], electronics [2,3,4], and biology [5]. The new applications require a better droplet deposition quality, which needs more precious control of droplet generation. In piezoelectric three-dimensional inkjet printing, droplets are generated by the deformation of piezoelectric ceramics. The expected printing effect can be achieved by matching material properties, printhead structures, and driving waveform. The driving waveform design is the only way to improve the droplet forming quality, as it is hard to adjust the printhead structure and material properties during the printing process. As most of the materials used in the applications above are liquids containing polymers, these materials have shearing-thinning viscosities [1,2,3,4,5], which affect the droplet generation [6]. Therefore, it is necessary to consider the shearing-thinning viscosities of materials when designing the driving waveform.

Currently, it is common to seek the driving waveforms through inkjet experiments. Single trapezoidal driving waveform (STDW) is the most common driving waveform to produce droplets [7,8]. Stable printing process can be obtained by adjusting the amplitude and duration of the driving waveform. Under the excitation of this kind of driving waveform, there will be not only main droplets but also satellite droplets, which is attributed to the residual fluid vibration inside the printhead during the printing process [9,10]. The phenomenon is common in power-law inkjet printing due to time-varying viscosities [1,3,4,5,6], which degrades the printing performance. To eliminate the residual fluid vibration inside the printhead, a double trapezoidal drive waveform (DTDW) is designed [7,8,9,10], of which the first trapezoidal pulse is used to form the main droplet and the second one is used to restore fluid to its original state inside the printhead. This method can reduce the satellite droplets, but there remain some low-energy residual vibrations [8,9], which still jeopardize the final printing quality. Smaller droplets could be produced by applying complex waveforms [11]. Computational fluid dynamics (CFD) is another way to find appropriate driving waveforms [12]. On some occasions, this method is viewed as an auxiliary means, which can help identify the range of parameters to decrease tests [13]. The driving waveform design methods from the above research are always empirical methods for specific fluids, which derive from many input–output data. It is hard to avoid waste in experiments and computation during early trials.

By contrast, the theoretical models have more effective instruction due to their universality [14]. The lump element model is a classical model to design the driving waveform [15]. While the current theoretical models take no account of the shear-thinning viscosities, the corresponding driving waveform designs are not suitable for power-law fluids.

Squeeze-mode inkjet printhead is driven by a circular piezoelectric ceramic pipe. This printhead is a typical kind of inkjet printhead, which works following the drop-on-demand principle. When the driving waveform is applied, the deformation caused by the inverse piezoelectric effect changes the fluid volume at the piezoelectric ceramic pipe, which generates pressure waves travelling in the fluid. The fluid at the nozzle is pushed outwards by the positive pressure waves. A droplet can be launched when the kinetic energy of the fluid there is large enough to overcome the surface tension. It has been proved that the meniscus movement determines the printing performance, which means the release and velocity of a droplet depends on the volume flow rate at the nozzle outlet [16]. Therefore, the improvement of droplet formation quality can be ensured by controlling the volume flow rate at nozzle outlet. In a previous study, we have presented an equivalent circuit model that reflects the flow state of power-law fluid during printing [17]. This model is available to obtain the volume flow rate of power-law fluid at the nozzle outlet, but the driving waveform is integrated into the state matrix of the model. In addition, this model needs to be modified before it is applied to design a driving waveform.

For a piezoelectric printhead, it is very suitable to use an iterative learning method to improve the droplet formation quality as the droplet ejection is executed repeatedly during material deposition. The input of the driving waveform at any time will affect the subsequent output as the power-law fluid printhead system is causal. PID iterative learning law is not suitable to obtain a sound driving waveform due to the fact that it only considers the system information at the last iteration cycle. Moreover, the power-law fluid printhead system is also nonlinear time varying, and it is necessary to establish the relationship between driving waveform and volume flow rate at the nozzle outlet.

The study aims to achieve the design of a piezoelectric printhead driving waveform for power-law fluid inkjet printing based on the iterative learning method. In Section 2, the equivalent circuit model in the previous study was modified and parameter estimation was presented. In Section 3, the causal relationship between the driving waveform and the volume flow rate at the nozzle outlet was established. The learning law of the iterative learning method was derived based on the gradient descent method. The convergence of iterative learning law was also demonstrated. In Section 4, experiments were carried out to prove the modified model and driving waveform design method. In Section 5, a summary is given.

## 2. Model Modification and Parameter Estimation

**Figure 1:**
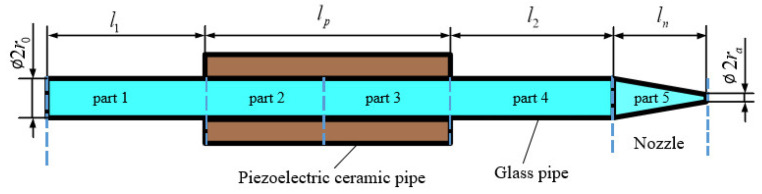
Structure and layout of the printhead.

The squeeze-mode printhead mentioned above mainly consists of a piezoelectric ceramic pipe and a glass pipe, of which one end is the nozzle. The piezoelectric ceramic pipe is an actuator, and the glass tube is full of fluid. According to the printhead working process described above, the deformation of piezoelectric ceramic pipe leads to a change in the inner fluid density, which makes fluid flow in the glass pipe. The printhead is divided according to flow states which vary with glass pipe structure and pressure wave propagation. The structure and layout of the printhead are illustrated in Figure 1.

To improve method reliability, it is preferable to apply the driving waveform design based on a model which can reflect the relationship between system input and output. In the previous study, an equivalent circuit model was presented to describe the flow state of the power-law fluid inside the printhead. The driving waveform changes the pipe volume, making fluid density change to drive the fluid flow. The volume change of pipe is equivalent to a time-varying capacitor, of which the expression is shown as [17]:

(1) $$C_{p}=\frac{l_{p}\pi \left[V_{e}{\left(t\right)}^{2}e^{2}-2r_{0}V_{e}\left(t\right)e\right]}{c^{2}\rho_{0}}$$

where *V_e_(t)* is the driving waveform, *e* is the conversion coefficient of the inverse piezoelectric effects [18], $\rho_{0}$ is the origin density, *c* is the sound velocity in fluid, and $l_{p}\pi \left[V_{e}{\left(t\right)}^{2}e^{2}-2r_{0}V_{e}\left(t\right)e\right]$ is the volume change ΔV. Equation (1) means the driving waveform is a part of a system state parameter, making it not convenient to design the controlling method. The model needs to be modified, and it is necessary to convert the driving waveform into system state variables such as electric current or voltage. The change in pipe diameter caused by the driving waveform is so tiny that the volume change ΔV could be simplified as follows:

(2) $$\Delta V=-2\pi rl_{p}eV_{e}\left(t\right)$$

The length to diameter ratio of pipe is so big that the mass conservation equation could be simplified as:

(3) $$\left(\rho_{0}-\Delta \rho \right)\left(V_{0}-\Delta V\right)=\rho_{0}V_{0}+\int_{0}^{t}\rho_{0}\pi r_{0}{}^{2}\left(u_{in}-u_{out}\right)dt$$

where Δρ means the density change, $V_{0}$ means the origin volume, $u_{in}$ and $u_{out}$ means the average inflow velocity and average outflow velocity in axial direction, respectively. By the further derivation, Equation (3) could be simplified as:

(4) $$\Delta \rho =-\frac{\rho_{0}}{V_{0}}\left(\Delta V+\int_{0}^{t}\Delta qdt\right),\;\Delta q=\pi r^{2}\left(u_{in}-u_{out}\right)$$

Equation (4) means the density change in the printhead is composed of pipe volume change and volume flow rate difference. In addition, the density change caused by the volume change could be converted into the volume flow rate, which is shown as:

(5) ∫Δqdt=−ΔV

Differentiate Equation (5) concerning time and obtain the electric current $i_{s}$ that represents the system input, which is shown as:

(6) $$i_{s}=2\pi rl_{p}e\frac{dV_{e}\left(t\right)}{dt}$$

**Figure 2:**
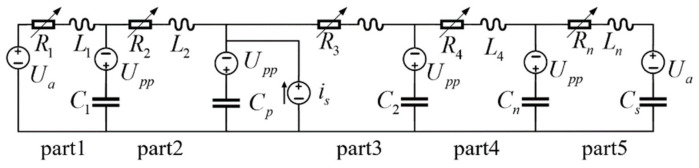
Circuit diagram of the modified model.

As the model is a lumped element model, all the physical parameters are concentrated. The fluid flow begins from the middle of the piezoelectric ceramic pipe, so $i_{s}$ works between part2 and part3 in Figure 2. Flow states change at the interfaces between parts, which embodies the fluid compressibility. The capacitor is the energy storage element in the circuit, representing fluid compressibility. Therefore, the capacitors work between parts in the equivalent circuit model, and the corresponding formulations are referred to [17]. There is no change in the resistors that represent fluid resistance, the inductors that represent fluid inertia and the initial voltage that represent the initial pressure and the surrounding pressure, which are the same as that in [17].

Combining the model modification with the derivation of the equivalent circuit model in [17], we obtained the circuit diagram that represents the modified model, which is shown in Figure 2.

The state equation of the above modified equivalent circuit model is obtained by using Kirchhoff’s voltage law (KVL) and Kirchhoff’s current law (KCL), which is shown as follows:

(7) $$\left\{\begin{array}{l} \dot{x}=Ax+Bu\\ y=Cx+Du \end{array}\right.$$

$$C=\left[\begin{array}{llllllllll} 0 & 1 & 0 & 0 & 0 & 0 & 0 & 0 & 0 & 0 \end{array}\right]\;D=\left[0\right]\;u={\left[\begin{array}{ll} U_{a}+U_{pp} & i_{s} \end{array}\right]}^{T}\;B={\left[\begin{array}{l} \begin{array}{llllllllll} 0 & \frac{1}{C_{p0}} & 0 & 0 & 0 & 0 & 0 & 0 & 0 & 0 \end{array}\\ \begin{array}{llllllllll} 0 & 0 & 0 & 0 & 0 & \frac{1}{L_{1}} & 0 & 0 & 0 & -\frac{1}{L_{n}} \end{array} \end{array}\right]}^{T}$$

$$A=\left[\begin{array}{cccccccccc} 0 & 0 & 0 & 0 & 0 & \frac{1}{C_{1}} & -\frac{1}{C_{1}} & 0 & 0 & 0\\ 0 & 0 & 0 & 0 & 0 & 0 & \frac{1}{C_{p0}} & -\frac{1}{C_{p0}} & 0 & 0\\ 0 & 0 & 0 & 0 & 0 & 0 & 0 & \frac{1}{C_{2}} & -\frac{1}{C_{2}} & 0\\ 0 & 0 & 0 & 0 & 0 & 0 & 0 & 0 & \frac{1}{C_{n}} & -\frac{1}{C_{n}}\\ 0 & 0 & 0 & 0 & 0 & 0 & 0 & 0 & 0 & \frac{1}{C_{s}}\\ -\frac{1}{L_{1}} & 0 & 0 & 0 & 0 & -\frac{R_{1}\left(i_{1}\left(t\right)\right)}{L_{1}} & 0 & 0 & 0 & 0\\ \frac{1}{L_{2}} & -\frac{1}{L_{2}} & 0 & 0 & 0 & 0 & -\frac{R_{2}\left(i_{2}\left(t\right)\right)}{L_{2}} & 0 & 0 & 0\\ 0 & \frac{1}{L_{3}} & -\frac{1}{L_{3}} & 0 & 0 & 0 & 0 & -\frac{R_{3}\left(i_{3}\left(t\right)\right)}{L_{3}} & 0 & 0\\ 0 & 0 & \frac{1}{L_{4}} & -\frac{1}{L_{4}} & 0 & 0 & 0 & 0 & -\frac{R_{4}\left(i_{4}\left(t\right)\right)}{L_{4}} & 0\\ 0 & 0 & 0 & \frac{1}{L_{n}} & -\frac{1}{L_{n}} & 0 & 0 & 0 & 0 & -\frac{R_{n}\left(i_{5}\left(t\right)\right)}{L_{n}} \end{array}\right]$$

The state equation shows the relationship between the driving waveform and volume flow rate at the nozzle outlet.

## 3. Iterative Learning Method

What the state equation above describes is a nonlinear time-varying system, of which the resistors represent the nonlinear time-varying viscosity of power-law fluid. For the convenience of the solution, the printhead system is discretized, and the discrete form [19] of the state equation can be written as:

(8) $$x\left(\left(n+1\right)T\right)=G_{n}x(nT)+TBu\left(nT\right),G_{n}=\left(I+A_{n}T\right)$$

where x(nT) is the system state variable at time *nT*, $G_{n}$ is the state-transition matrix at time *nT*, I is the unit matrix, $A_{n}$ is the state matrix at time *nT* in which the resistors are acquired by plugging the current values of x(nT), T is the discrete time step, B is the input matrix, and $u\left(nT\right)$ is the system input at time *nT*, which is composed of $i_{s}\left(nT\right)$ and $U_{a}+U_{pp}$. Divide the time interval into N steps, and all the system state variables within the time interval are shown as:

$$\left[\begin{array}{c} x(T)\\ x(2T)\\ \vdots \\ x\left((N-1)T\right)\\ x(NT) \end{array}\right]=\left[\begin{array}{c} TB\\ TG_{2}B\\ \vdots \\ TG_{2}G_{3}\cdots G_{N-1}B\\ TG_{2}G_{3}\cdots G_{N}B \end{array}\begin{array}{c} 0\\ TB\\ \vdots \\ TG_{3}G_{4}\cdots G_{N-1}B\\ TG_{3}G_{4}\cdots G_{N}B \end{array}\begin{array}{c} 0\\ 0\\ \vdots \\ TG_{4}G_{5}\cdots G_{N-1}B\\ TG_{4}G_{5}\cdots G_{N}B \end{array}\begin{array}{c} \cdots \\ \cdots \\ \cdots \\ \cdots \\ \cdots \end{array}\begin{array}{c} 0\\ 0\\ \vdots \\ TB\\ TG_{N}B \end{array}\begin{array}{c} 0\\ 0\\ \vdots \\ 0\\ TB \end{array}\right]\left[\begin{array}{c} u(0)\\ u(T)\\ \vdots \\ u\left((N-2)T\right)\\ u((N-1)T) \end{array}\right]\;+\left[\begin{array}{c} G_{1}\\ G_{1}G_{2}\\ \vdots \\ G_{1}G_{2}\cdots G_{N-1}\\ G_{1}G_{2}\cdots G_{N} \end{array}\right]x\left(0\right)$$

The system input is composed of a driving waveform and external pressure. The driving waveform is time varying while external pressure is constant. Therefore, the system state variables can be split into two parts, which is written as:

$$\left[\begin{array}{c} x(T)\\ x(2T)\\ \vdots \\ x\left((N-1)T\right)\\ x(NT) \end{array}\right]=\left[\begin{array}{c} TB_{1}\\ TG_{2}B_{1}\\ \vdots \\ TG_{2}G_{3}\cdots G_{N-1}B_{1}\\ TG_{2}G_{3}\cdots G_{N}B_{1} \end{array}\begin{array}{c} 0\\ TB_{1}\\ \vdots \\ TG_{3}G_{4}\cdots G_{N-1}B_{1}\\ TG_{3}G_{4}\cdots G_{N}B_{1} \end{array}\begin{array}{c} 0\\ 0\\ \vdots \\ TG_{4}G_{5}\cdots G_{N-1}B_{1}\\ TG_{4}G_{5}\cdots G_{N}B_{1} \end{array}\begin{array}{c} \cdots \\ \cdots \\ \cdots \\ \cdots \\ \cdots \end{array}\begin{array}{c} 0\\ 0\\ \vdots \\ TB_{1}\\ TG_{N}B_{1} \end{array}\begin{array}{c} 0\\ 0\\ \vdots \\ 0\\ TB_{1} \end{array}\right]\left[\begin{array}{c} u_{1}\left(0\right)\\ u_{1}\left(T\right)\\ \vdots \\ u_{1}\left((N-2)T\right)\\ u_{1}\left(\left(N-1\right)T\right) \end{array}\right]+\;\left[\begin{array}{c} TB_{2}\\ TG_{2}B_{2}\\ \vdots \\ TG_{2}G_{3}\cdots G_{N-1}B_{2}\\ TG_{2}G_{3}\cdots G_{N}B_{2} \end{array}\begin{array}{c} 0\\ TB_{2}\\ \vdots \\ TG_{3}G_{4}\cdots G_{N-1}B_{2}\\ TG_{3}G_{4}\cdots G_{N}B_{2} \end{array}\begin{array}{c} 0\\ 0\\ \vdots \\ TG_{4}G_{5}\cdots G_{N-1}B_{2}\\ TG_{4}G_{5}\cdots G_{N}B_{2} \end{array}\begin{array}{c} \cdots \\ \cdots \\ \cdots \\ \cdots \\ \cdots \end{array}\begin{array}{c} 0\\ 0\\ \vdots \\ TB_{2}\\ TG_{N}B_{2} \end{array}\begin{array}{c} 0\\ 0\\ \vdots \\ 0\\ TB_{2} \end{array}\right]\left[\begin{array}{c} u_{2}\left(0\right)\\ u_{2}\left(T\right)\\ \vdots \\ u_{2}\left((N-2)T\right)\\ u_{2}\left(\left(N-1\right)T\right) \end{array}\right]\;+\left[\begin{array}{c} G_{1}\\ G_{1}G_{2}\\ \vdots \\ G_{1}G_{2}\cdots G_{N-1}\\ G_{1}G_{2}\cdots G_{N} \end{array}\right]x\left(0\right)$$

$$B_{1}={\left[\begin{array}{llllllllll} 0 & \frac{1}{C_{p0}} & 0 & 0 & 0 & 0 & 0 & 0 & 0 & 0 \end{array}\right]}^{T},B_{2}={\left[\begin{array}{llllllllll} 0 & 0 & 0 & 0 & 0 & \frac{1}{L_{1}} & 0 & 0 & 0 & -\frac{1}{L_{n}} \end{array}\right]}^{T}$$

$$\left[\begin{array}{l} u_{1}(0)\\ u_{1}(T)\\ \vdots \\ u_{1}\left((N-2)T\right)\\ u_{1}((N-1)T) \end{array}\right]=\left[\begin{array}{l} i_{s}(0)\\ i_{s}(T)\\ \vdots \\ i_{s}\left((N-2)T\right)\\ i_{s}((N-1)T) \end{array}\right],\left[\begin{array}{l} u_{2}(0)\\ u_{2}(T)\\ \vdots \\ u_{2}\left((N-2)T\right)\\ u_{2}((N-1)T) \end{array}\right]=\left[\begin{array}{l} U_{a}+U_{pp}\\ U_{a}+U_{pp}\\ \vdots \\ U_{a}+U_{pp}\\ U_{a}+U_{pp} \end{array}\right]$$

where $B_{1}$ is the input matrix corresponding to the driving waveform, $B_{2}$ is the input matrix corresponding to external pressure. From the above derivation, the system is converted into a single input single output (SISO) system. In addition, the relationship between system input and system output at time *nT* is shown as:

(9) $$\begin{array}{l} y(nT)=C[G_{1}G_{2}\cdots G_{n}x(0)+TG_{2}G_{3}\cdots G_{n}B_{1}u_{1}(0)+\\ TG_{3}G_{4}\cdots G_{n}B_{1}u_{1}(T)+TG_{4}G_{5}\cdots G_{n}B_{1}u_{1}(2T)+\cdots +\\ TG_{n}{Β}_{1}u_{1}\left((n-2)T\right)+TB_{1}u_{1}((n-1)T)+TG_{2}G_{3}\cdots G_{n}B_{2}u_{2}(0)+\\ TG_{3}G_{4}\cdots G_{n}B_{2}u_{2}(T)+TG_{4}G_{5}\cdots G_{n}B_{2}u_{2}(2T)+\cdots +\\ TG_{n}B_{2}u_{2}\left((n-2)T\right)+TB_{2}u_{2}((n-1)T)] \end{array}$$

The input of the driving waveform at any time will affect the subsequent output as the power-law fluid printhead system is causal. It is not easy to achieve the expected effect through the classical PID iterative learning law. In contrast, it is more suitable to use the gradient descent method to design the iterative learning law. In addition, the power-law fluid printhead system is a nonlinear time-varying system, and the state matrix is constant only at the same time. To ensure convergence of the iterative process within the time interval, the iteration learning control is required to realize to be convergent at each step. The discrete form of system reference output is defined as:

(10) $$y_{d}={\left[y_{d}(T),y_{d}(2T),\cdots ,y_{d}(NT)\right]}^{T}$$

For a printhead system, the goal of the iterative learning control waveform design method is to find a system input *u* within a given time interval [0 *NT*] so that the system output is consistent with the reference output at each time step. The convergent learning law makes the system output close to the reference output as the iterative step *k* increases. The system output will coincide with the reference output when *k* approaches infinity. The error *e(nT, k)* is the difference value between the system output and reference output corresponding to iteration *k* at time *nT,* which is written as:

(11) $$e(nT,k)=y_{d}\left(nT\right)-y(nT,k)$$

The quadratic form of the error corresponding to iteration *k* at time *nT* can be written as:

(12) $$E(nT,k)=\frac{1}{2}e{\left(nT,k\right)}^{2}$$

where E(nT,k) is the absolute error corresponding to iteration *k* at time *nT*. The purpose of the iterative learning waveform design is to find a system input $u_{1}\left(\left(n-1\right)T\right)$ to make $\underset{k\to \infty }{\lim}E\left(nT,k\right)=0$. To rapidly reduce the absolute error *E*, the steepest descent method is used to design iterative learning laws. The negative gradient of the absolute error corresponding to iteration *k* at time *nT* can be written as:

(13) $$v\left(nT,k\right)=-\frac{dE(nT,k)}{du_{1}(\left(n-1\right)T,k)}$$

Therefore, the learning law of the iterative learning system is shown as follows.

(14) $$u_{1}\left(\left(n-1\right)T,k+1\right)=u_{1}\left(\left(n-1\right)T,k\right)+\eta \left(nT,k\right)v\left(nT,k\right)$$

where $\eta \left(nT,k\right)$ is the iterative step length corresponding to iteration *k* at time *nT*.

The convergent iterative learning law implies that the inequality *E(nT, k)* > *E(nT, k + 1)* is true. The error corresponding to iteration *k*+1 at time *nT* can be written as:

(15) $$e(nT,k+1)=e(nT,k)-\eta \left(nT,k\right)v\left(nT,k\right)$$

The absolute error corresponding to iteration *k+1* at time *nT* can be written as:

(16) $$E(nT,k+1)=E(nT,k)+\frac{1}{2}\left[{\left(\eta \left(nT,k\right)v\left(nT,k\right)\right)}^{2}-2e(nT,k)\eta \left(nT,k\right)v\left(nT,k\right)\right]$$

The second term on the right-hand side of Equation (16) is denoted as the absolute error increment Δ*E(nT, k)* corresponding to iteration *k* at time *nT*. The expression for the absolute error increment Δ*E(nT, k)* can be written as:

(17) $$\Delta E(nT,k)=\frac{1}{2}{\left(v\left(nT,k\right)\right)}^{2}\left[{\left(\eta \left(nT,k\right)\right)}^{2}-\frac{2e(nT,k)}{v\left(nT,k\right)}\eta \left(nT,k\right)\right]$$

To reduce the absolute error *E* at each iteration step, the absolute error increment Δ*E* < 0 is required at each iteration step. When the negative gradient $v\left(nT,k\right)$ is not zero, Equation (17) is a quadratic function of the iteration step length $\eta \left(nT,k\right)$. When the error *e(nT, k)* is not zero, the absolute error increment Δ*E* must have a negative interval. In addition, there is always an $\eta \left(nT,k\right)$ to make the absolute error increment Δ*E* negative. When the iteration step length $\eta \left(nT,k\right)$ is $\frac{e(nT,k)}{v\left(nT,k\right)}$, the absolute error increment Δ*E* is minimal. To summarize, the iterative learning law presented in this study is convergent.

## 4. Results and Discussion

**Figure 3:**
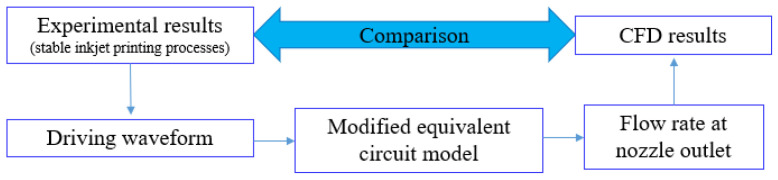
Verification approach of modified model.

To prove the modified equivalent circuit model and the driving waveform design method based on iterative learning control, experiments should be carried out to track the volume flow rate at the nozzle outlet. While there is no direct way to detect the volume flow rate, it is much easier to detect the drop formation through the droplet watch system [7,8,9], and there has been much research about the simulation of drop formation through CFD [12,13]. Therefore, it is viable to verify the modified model and the driving waveform design method by comparing CFD results and experimental results. To ensure the reliability and clearness of the comparison, we chose the stable inkjet printing processes as the study object. The modified model is effective when the experimental results are consistent with the CFD results, of which the boundary conditions are the volume flow rates obtained from the modified model. The verification approach of modified model is shown in Figure 3.

**Figure 4:**
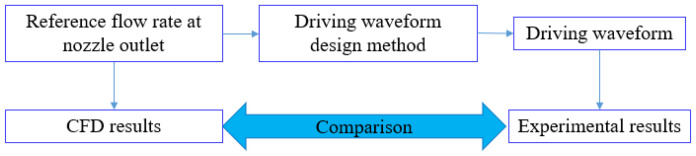
Verification approach of driving waveform design method.

The driving waveform design method is feasible when the experimental results caused by the modified driving waveforms are consistent with the CFD results, of which the boundary conditions are the reference flow rates. The verification approach of driving waveform design method is shown in Figure 4.

As xanthan gum solution has been chosen to study the generation of the power-law fluid droplet in [2], in this study, xanthan gum solution was produced to verify the above points, of which the concentration was 0.2 g/L. The deionized (DI) water was chosen as the reference substance as its viscosity is constant.

**Figure 5:**
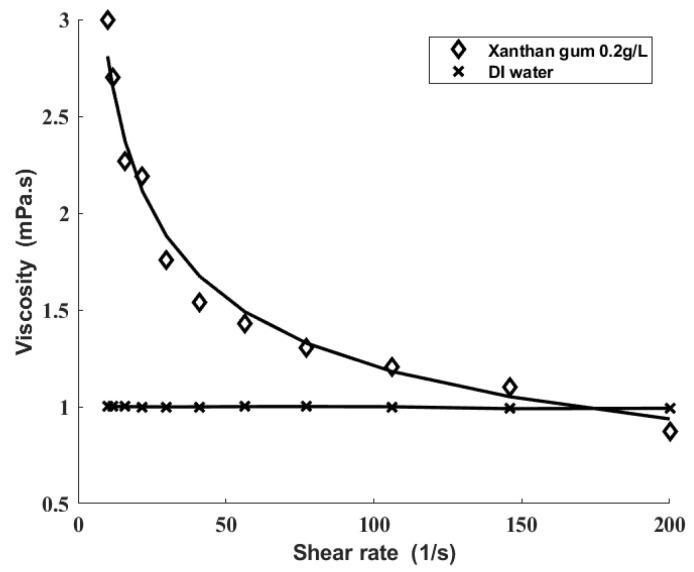
Viscosities of xanthan gum solution and DI water. The measured values are marked by symbols. The fitting curves are marked by solid lines.

The fluid viscosities were measured by a visualization rheometer (MCR302, Anton Paar, Graz, Austria), and the power-law model shown as (18) was used to fit the fluid viscosities.

(18) $$\eta =\mu {\left(\dot{\gamma }\right)}^{n-1}$$

where η is the shear viscosity, μ is the viscosity factor, n is the power-law factor, and $\dot{\gamma }$ is the shear rate. The measured results are shown in Figure 5.

The viscosity factor of the prepared solution was 6.536. The power-law factor of the prepared solution was 0.6334. The surface tension of the prepared solution was measured by the Du Nouy ring method (DCAT25 Tensiometer, DataPhysics Instruments, Stuttgart, Germany), of which the value was 68.3 mN/m.

The xanthan gum concentration was so small that there was no need to take the effect of xanthan gum on the density of the solution into consideration. The solution density was the same as that of DI water, and the acoustic speed inside the solution was consistent with that in DI water.

**Figure 6:**
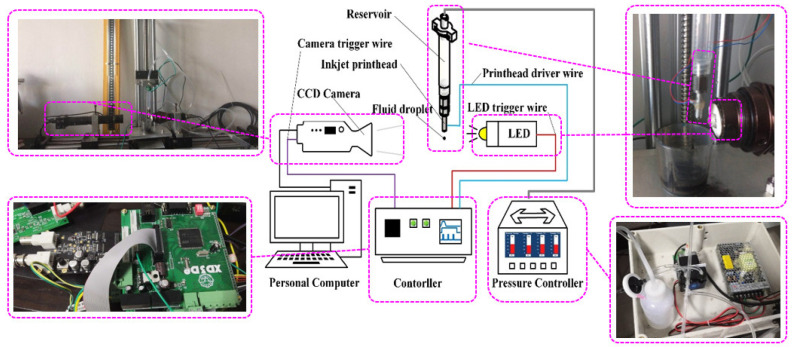
Schematic of the experimental system.

The droplet watch system in [20] was established to obverse the droplet formation, which is shown in Figure 6. The system mainly consists of a CCD camera, LED strobe, printhead, droplet watch system controller (DWSC), pressure controller, and a data processing software that runs on a PC. The CCD camera (MVD040SM, Microvision) with an external trigger interface is used to record the droplet images at different times. The LED strobe is used to control the exposure time because there is insufficient incident light. The exposure time of CCD camera can be adjusted by setting the lighting time of the LED strobe. To make the edge of the droplet image captured by the CCD camera as clear as possible without reducing the contrast, the lighting time of the LED strobe should be as short as possible. The pressure controller generates a negative pressure inside the printhead to balance the weight of the fluid. The data processing software is used to analyze photos captured by the CCD camera and extract the droplet formation.

The squeeze-mode piezoelectric printhead in [17] was used to eject droplets, of which the dimension is listed in Table 1. 

**Figure 7:**
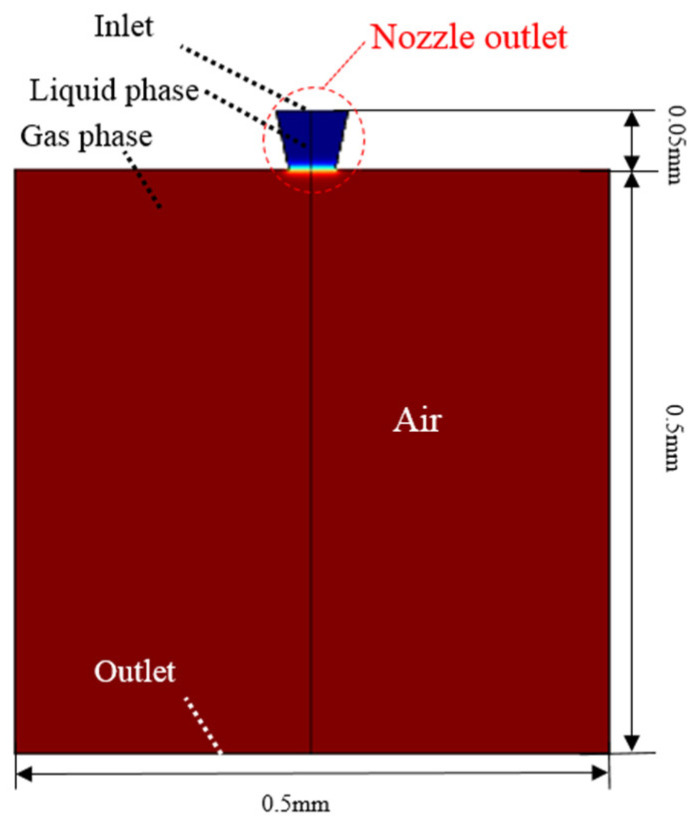
CFD two-phase simulation.

The CFD two-phase simulation was used to simulate the droplet generation as shown in Figure 7. In the present research, the area of CFD simulation included the nozzle outlet and the air nearby. This simulation consisted of two modules: level set and laminar flow. Level set module was used to present the change of liquid phase and gas phase. The nozzle outlet was set as liquid phase and the air nearby was set as gas phase. Laminar flow module was used to present the motion characteristic of fluid. The upper boundary of the nozzle outlet was set as the inlet of fluid, where the boundary condition was the flow rates calculated by the revised model. The lower boundary of the air nearby was set as the outlet of fluid, where the boundary condition was zero-pressure.

**Figure 8:**
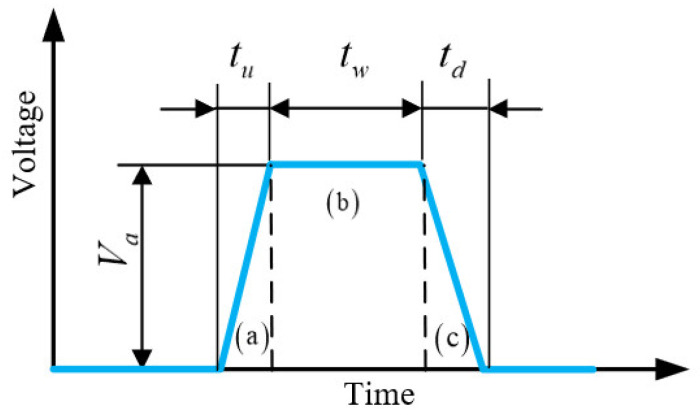
Diagram of single trapezoidal driving waveform.

In the primary stage of the experiment, an origin driving waveform was determined from trials and errors [21], which could lead to a stable inkjet printing process. A single trapezoidal driving waveform was used as shown in Figure 8. During the rising edge (a), the piezoelectric tube expands to draw ink from the reservoir and the fluid inflow continues during the dwell time (b). During the falling edge (c), the piezoelectric tube shrinks, and the fluid is squeezed out. The printing processes are determined by the time intervals and the wave amplitude. In the present research, the rising and falling edges of the driving waveform were in the range of 1–5 μs. The dwell time was in the range of 4–32 μs, and the wave amplitude was in the range of 12–50 v. We adjusted the characteristics of the single trapezoidal driving waveform to obtain a stable inkjet printing process. The corresponding characteristics of the origin waveform are listed in Table 2.

**Figure 9:**
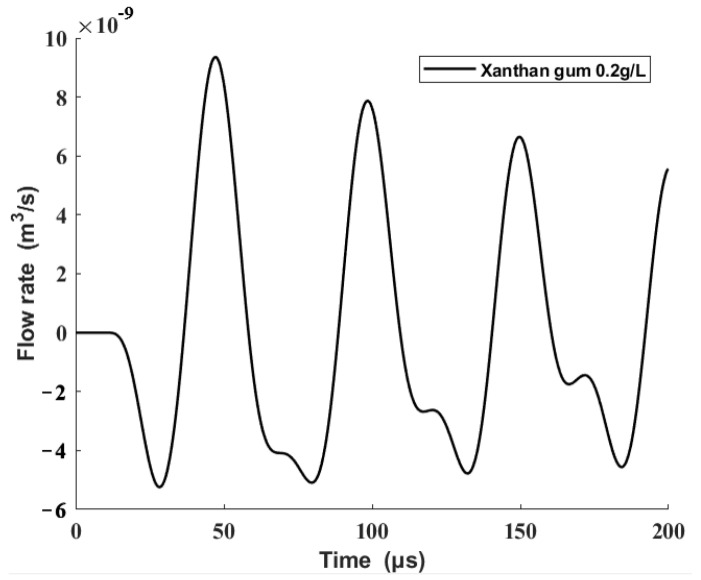
Result of the modified model.

Then, the origin driving waveform was taken into the modified equivalent circuit model in this paper to obtain the volume flow rate at the nozzle outlet, which is shown in Figure 9.

**Figure 10:**
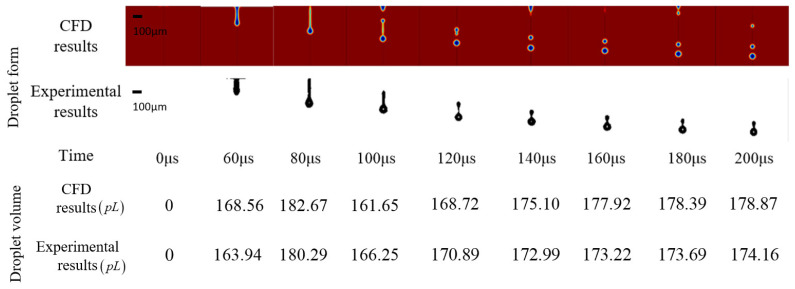
Comparison of xanthan gum solution CFD result and experimental result at the condition of the origin driving waveform.

The above result was set as the boundary condition of the CFD two-phase fluid model to simulate the drop formation at the nozzle. The experimental result was consistent in time, droplet form, and droplet volume with the CFD result, which proves the modified model. The comparison is shown in Figure 10.

**Figure 11:**
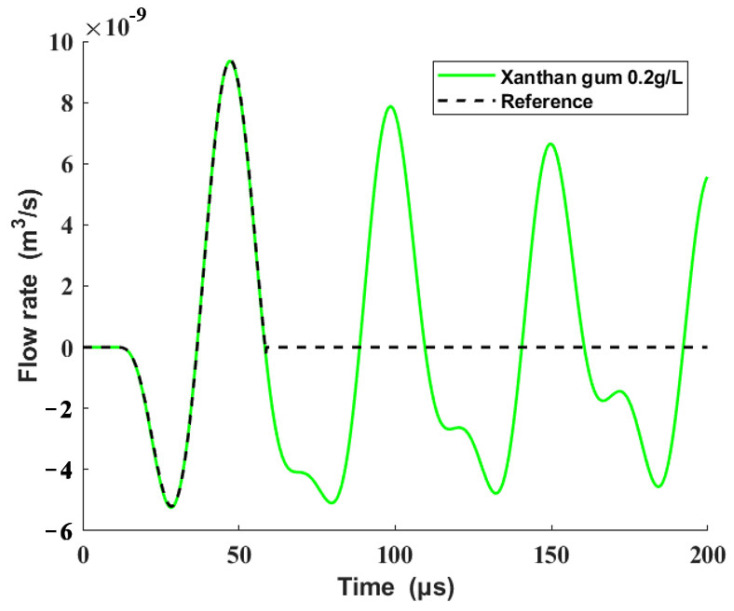
Origin volume flow rates and the reference output.

It is resulting flow rates, except for the initial crest, which push the fluid outward to form droplets that cause residual oscillations at the outlet of the nozzle [7]. To reduce the satellite droplets and eliminate the residual oscillations [8,9,10], the volume flow rates after the first wave peak are eliminated, and the remaining volume flow rate curve is used as the reference output. Figure 11 shows the comparison between the origin volume flow rates and reference output.

**Figure 12:**
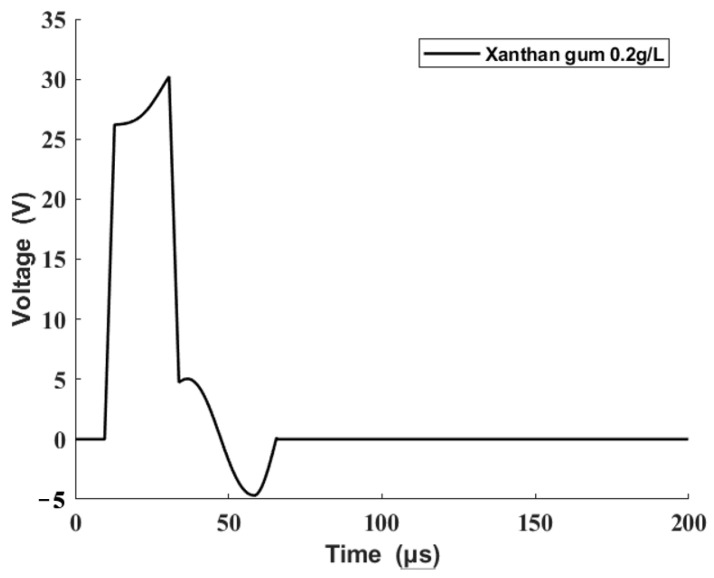
Modified driving waveform.

The reference output was taken into the driving waveform design method presented in this study to obtain the modified driving waveform, which is shown in Figure 12.

**Figure 13:**
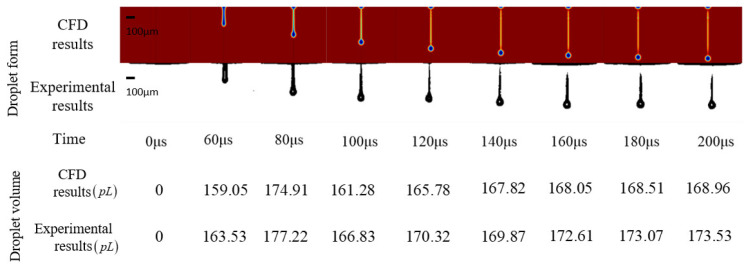
Comparison of xanthan gum solution CFD result and experimental result at the condition of the modified driving waveform.

The modified driving waveform was used to carry out the droplet formation experiment, and the reference output was used as the boundary condition of the CFD two-phase fluid model. The experimental result and CFD result were consistent in time, droplet form, and droplet volume, which proves the driving waveform method which is shown in Figure 10. The comparison is shown in Figure 13.

## 5. Conclusions

This paper proposes a waveform design method for power-law fluid piezoelectric printing. The equivalent circuit model that describes the fluid flow inside the printhead was modified to obtain the relationship between the driving waveform and volume flow rate, which provide the basis for iterative learning control. A new iterative learning law that minimizes the error was designed to ensure that the iterative learning process is convergent. The comparisons between experimental results and CFD results indicate that the modified model was effective, and the proposed driving waveform design method can control the volume flow rate of the fluid at the nozzle, which lays the foundation for the formation of single and stable droplets during the power-law fluid printing process. However, the breakup of the fluid column and the generation of droplet are complex, and different volume flow rates will lead to different inkjet printing performance. For more complex applications, it is difficult to find a corresponding reference flow rate, which also needs further research.

## Figures and Tables

**Table 1:** Structure parameters of printhead.

Length (mm)	$l_{1}$	$l_{2}$	$l_{3}$	$l_{4}$	$r_{0}$	$r_{a}$
	8.87	8.2	4.71	1	0.235	0.04

**Table 2:** Characteristics of the origin driving waveform.

	$V_{a}\left(v\right)$	$t_{u}\left(\mu s\right)$	$t_{w}\left(\mu s\right)$	$t_{d}\left(\mu s\right)$
xanthan gum 0.2 g/L	26	3	18	3
